# Unusual liver‐related heart injury

**DOI:** 10.1002/jgh3.12373

**Published:** 2020-06-12

**Authors:** Riccardo Nevola, Valerio Rosato, Pasquale Perillo, Nicolino Esposito, Domenico Massa, Anna Maria Frascino, Vittorio Capurro, Ernesto Claar

**Affiliations:** ^1^ Internal Medicine and Hepatology Unit Betania Evangelical Hospital Naples Italy; ^2^ Department of Advanced Medical and Surgical Sciences University of Campania “Luigi Vanvitelli Naples Italy; ^3^ Hypertension Research Center, UOC Emergency Medicine Federico II University Hospital Naples Italy

**Keywords:** cardiac tamponade, liver cysts, polycystic liver disease, shock

## Abstract

A 72‐year‐old man with polycystic liver disease and unexplained shock was admitted to our Emergency Department. The presence of turgidity in the jugular veins and acute prerenal kidney failure led to a possible hypothesis of right ventricular heart failure. A massive hepatic cyst resulted in right atrial compression and, secondarily, a state of shock. Surgical decompression by drainage of the hepatic cyst resulted in rapid improvement in the patient's hemodynamics. We report the description of an extremely rare complication of polycystic liver disease.

## Graphical abstract

Polycystic liver disease (PCLD) is a rare autosomal‐dominant genetic condition characterized by the presence of multiple cysts in the liver tissue. When extremely massive, the induced mass effect can lead to abdominal distension, gastroesophageal reflux and/or dyspnoea. This is the description of an extremely rare manifestation of the disease: cardiac tamponade.

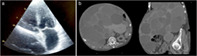

## Introduction

Polycystic liver disease is a rare autosomal‐dominant genetic condition characterized by the presence of multiple biliary cysts in the liver tissue.^1^ Most cases of PCLD have been associated with mutations in one of the following genes: (I) SEC63 (involved in the mechanisms of protein translocation in the endoplasmic reticulum) or (II) PRKCSH (which is a code for a protein named hepatocystin) genes. These mutations are able to induce the separation of ductal structures from the biliary tree, with secondary formation of hepatic cysts. Although these alterations occur at a very early stage, the disease often remains asymptomatic till the growth of the cysts becomes massive, more frequently in adulthood.^2^ When extremely massive, the induced mass effect can lead to abdominal distension, gastroesophageal reflux, and/or dyspnea. Hence, we report the description of an extremely rare manifestation of the disease: cardiac tamponade.

## Case Report

**Figure 1:**
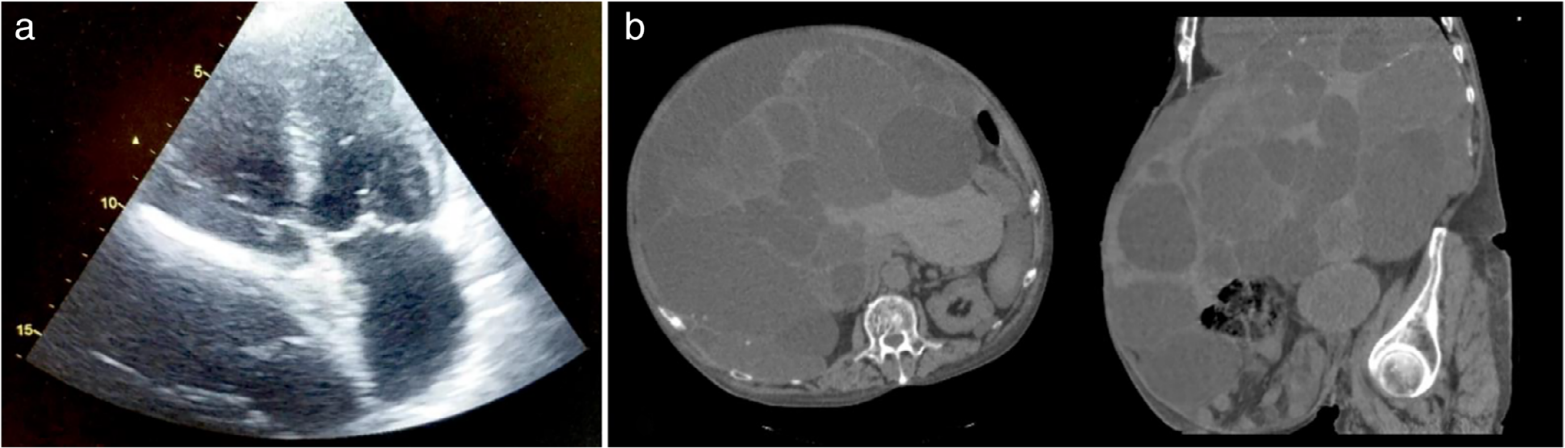
(a) Bed‐side cardiac ultrasound (apical 4‐chamber) showed a right atrial compression, caused by massive liver cysts; (b) computed tomography showed the presence of multiple liver cysts and their mass effect.

A 72‐year‐old man was admitted to the Emergency Department a few days after the onset of severe asthenia and remarkable dyspnea. On examining his medical history, the patient reported arterial hypertension under pharmacological treatment (discontinued approximately 3 days before the admission) and PCLD. On physical examination, the patient showed hypotension (blood pressure: 70/40 mmHg) and tachycardia (heart rate: 112 bpm); peripheral oxygen saturation and body temperature were instead within the reference ranges. Furthermore, a spherical abdomen, tense but treatable, was observed, as well as massive liver‐related cysts, detectable both by observation and palpation across all the abdominal quadrants. Clinical cardiothoracic examination was normal. Skin and mucous membranes appeared normally hydrated. A slight turgidity in the jugular veins was also observable. The patient's arterial blood gas showed metabolic acidosis (pH: 7.28, HCO_3_^−^ 14 mmol/L) with an increase in lactates (3.7 mmol/L). Infusions of crystalloids did not produce any improvement in the mean blood pressure. Significant modifications in the blood test were also observed: increased levels of creatinine (Cr: 3.36 mg/dL) and serum blood urea (BUN—blood urea nitrogen 287 mg/dL), with a BUN/Cr ratio exceeding 85, suggestive of prerenal kidney failure. In addition, neutrophilic leukocytosis (WBC: 13.1 × 10^3^/μL), an increase in transaminases and in C‐reactive protein levels (CRP: 180.8 mg/L), and hyperkalemia (K: 7.22 mEq/L) were observed. Hence, the diagnostic hypotheses were as follows: septic shock (in favor of the hypothesis of high CRP and neutrophilic leukocytosis), hypovolemic/hemorrhagic shock (high BUN/Cr ratio), and cardiac tamponade/massive pulmonary embolism (jugular turgidity). A rapid cardiac ultrasound examination ruled out pericardial effusion (cardiac tamponade) and/or dilation of the right heart chambers (massive pulmonary embolism). However, right atrial compression was observed, caused by the massive liver cysts (Fig. 1a). Such a condition leads to an insufficient right ventricular filling with subsequent reduced right ventricular output and, in addition, reduced left ventricular output. It was also reasonable to suspect a compression of the inferior vena cava. In order to exclude abdominal compartment syndrome (ACS), urinary manometry was also performed, as an indirect estimation of the intra‐abdominal pressure, by using a Foley catheter. Our findings suggested intra‐abdominal hypertension (IAH) in the absence of compartment syndrome. Therefore, the final diagnosis was: “shock due to ab‐extrinsic cardiac tamponade caused by liver cysts, complicated by acute kidney failure with metabolic acidosis and hyperkalaemia.” The patient underwent an abdominal CT scan without contrast, as surgical evaluation (Fig. 1b), and was promptly transferred to the operating room for ultrasound‐guided drainage of the liver cysts. The drainage led to a rapid improvement of overall clinical conditions, blood pressure rapidly returned to within normal ranges, and the degree of kidney damage also gradually improved. Further elective percutaneous drainage procedures were performed, with the removal of about 30 liters of gall fluid. Despite the benign clinical course, the patient died a few weeks later due to complications related to septic shock.

## Discussion

In PCLD, the presence of multiple liver cysts is frequently asymptomatic. When the mass effect becomes relevant, abdominal distension, early satiety, or dyspnea may appear. Cardiac tamponade and subsequent shock are extremely rare complications, which require a high index of suspicion in order to be diagnosed. Conditions such as pulmonary embolism or severe pericardial effusion may result in a similar symptomatic set. Physical examinations, along with blood and instrumental tests, are essential to rule out more frequent causes of shock and likely confirm an extremely rare diagnosis such as cardiac tamponade secondary to massive liver cyst. At present, according to our evidence, only a single analogous case has been documented in the literature.^3^ On the other hand, some cases of ab extrinsic cardiac tamponade due to either massive ascites^4^ or hepatic subcapsular hematoma^5^ have been reported.

## Declaration of conflict of interest

None
